# A Novel Real-Time Path Servo Control of a Hardware-in-the-Loop for a Large-Stroke Asymmetric Rod-Less Pneumatic System under Variable Loads

**DOI:** 10.3390/s17061283

**Published:** 2017-06-04

**Authors:** Hao-Ting Lin

**Affiliations:** Department of Mechanical and Computer-Aided Engineering, Feng Chia University; Taichung 407, Taiwan; haotlin@fcu.edu.tw; Tel.: +886-4-2451-7250 (ext. 3525)

**Keywords:** rod-less pneumatic cylinder, asymmetrical load, fourier series approximation technique, path tracking servo control, hardware-in-the-loop

## Abstract

This project aims to develop a novel large stroke asymmetric pneumatic servo system of a hardware-in-the-loop for path tracking control under variable loads based on the MATLAB Simulink real-time system. High pressure compressed air provided by the air compressor is utilized for the pneumatic proportional servo valve to drive the large stroke asymmetric rod-less pneumatic actuator. Due to the pressure differences between two chambers, the pneumatic actuator will operate. The highly nonlinear mathematical models of the large stroke asymmetric pneumatic system were analyzed and developed. The functional approximation technique based on the sliding mode controller (FASC) is developed as a controller to solve the uncertain time-varying nonlinear system. The MATLAB Simulink real-time system was a main control unit of a hardware-in-the-loop system proposed to establish driver blocks for analog and digital I/O, a linear encoder, a CPU and a large stroke asymmetric pneumatic rod-less system. By the position sensor, the position signals of the cylinder will be measured immediately. The measured signals will be viewed as the feedback signals of the pneumatic servo system for the study of real-time positioning control and path tracking control. Finally, real-time control of a large stroke asymmetric pneumatic servo system with measuring system, a large stroke asymmetric pneumatic servo system, data acquisition system and the control strategy software will be implemented. Thus, upgrading the high position precision and the trajectory tracking performance of the large stroke asymmetric pneumatic servo system will be realized to promote the high position precision and path tracking capability. Experimental results show that fifth order paths in various strokes and the sine wave path are successfully implemented in the test rig. Also, results of variable loads under the different angle were implemented experimentally.

## 1. Introduction

The rapid development of high technology industries has resulted in increasing needs for precision positioning and high-speed actuation facilities in the semiconductor, manufacturing, and biomedical engineering industries. At present, these industries must urgently develop highly precise and highly responsive manufacturing and medical facilities. In recent years, the manufacturing industry has emphasized high precision and high efficiency by replacing manual labor in mass production with robot equipment. These robots are mostly actuated by fully developed motor systems, but they tend to impose constraints on assembly spaces within factories. Thus, additional space is required for the multiaxial assembly operations performed by such actuators.

Pneumatic drives are used to actuate various types of systems with pressure energy supplied by compressed air. Compared with hydraulic drives and motors, pneumatic drives are relatively fast, simply structured, clean, light, accessible, safe, easy to maintain, and responsive. They can be widely applied to industries that involve automation systems, semiconductors, photoelectric facilities, or medical equipment. However, pneumatic systems have several drawbacks that may increase the difficulty of pneumatic system control, including high compressibility, low rigidity, leakage, high nonlinearity, dead bands, and zero drift of the servo valves. Conventional pneumatic systems are mostly applied to point-to-point sequential control. The rapid development of precision technologies has caused managers to regard conventional open-loop control systems as technically inadequate; pneumatic systems must integrate sensors and closed-loop control to keep pace with the trends of high speed, high precision, and high quality in the manufacturing of medical equipment and other modern products. Therefore, vast quantities of data collected from measurement systems can be used for analysis and operation control purposes by a control unit to drastically improve pneumatic system performance.

Recent technological developments have led to several breakthroughs in pneumatic control that have gradually solved the problem of nonlinearity in pneumatic systems. The concept of nonlinearity in pneumatic systems was proposed by Shearer in 1954. To date, numerous scholars have created comprehensive models for nonlinearity in pneumatic systems. In the 1980s, relevant software and hardware technologies gradually matured and led to research in the servo control of pneumatic systems. In 1984, Weston et al. [1] applied feedback compensation to the control of pneumatic systems but overlooked interference effects. In 1987, Noritsugu [2,3] used proportional-integral-derivative controllers and pulse width modulation (PWM) to improve the speed and location control of pneumatic systems. In 1994, Sheu divided positioning control into two stages (i.e., speed control and position control) and achieved a precision of five micrometers in a no-load state. In 1997, Song and Ishida [4] treated pneumatic systems as two-stage systems, defined the boundaries of system uncertainty, and applied sliding mode control to increase the robustness of pneumatic servo systems. In 1998, Shih and Ma [5] employed fuzzy control to control the position of a pneumatic rod-less cylinder and used a modified differential PWM method to ameliorate the delay and hysteresis problems of conventional differential PWM methods. In 1999, Luor used an online learning neuro-fuzzy controller that utilized normalized scale factors to control the position of a pneumatic cylinder and achieved a precision of five or less micrometers in a no-load state. In 2000, Su and Kuo [6] achieved discontinuous variable structure control by integrating two sliding surfaces and thereby solved the problem of mismatch interference. In 2002, Ning and Bone [7] incorporated position velocity acceleration control with frictional compensation to improve the steady-state error of positioning in pneumatic systems and achieved a steady-state error of 10 µm for both vertical and horizontal movements. In 2004, Somyot and Manukid [8] proposed a genetic algorithm-based method incorporating H-inf control for pneumatic servo systems, revealing that system stability and robustness can be improved. In 2004, Chang applied a proportional-derivative (PD) controller and deadzone compensation to control a three-axis servo pneumatic system, achieving a positioning accuracy of one micrometer; in addition, a velocity feed-forward compensator was employed to improve the effects of linear tracking. In 2004, Chen and Hwang [9] used proportional derivative iterative learning controllers to control a pneumatic XY table system; the controllers were able to effectively track a given trajectory and could reject disturbances. In 2005, Cheng employed fuzzy sliding mode controllers with loading compensators to control the servo positioning control system of a vertical pneumatic cylinder and achieved a positioning accuracy of 100 nm under various load conditions. In 2005, Ning et al. [10] proposed a nonlinear dynamic model for pneumatic servo systems that included equations of pneumatic cylinder dynamics, load motion, friction and valve characteristics; the model was able to predict the positions of pneumatic cylinder pistons and the pressure levels of air chambers in pneumatic cylinders. In 2007, Liu applied the function approximation technique to an adaptive sliding controller to control a three-axial pneumatic servo system and achieve a positioning accuracy of one micrometer, attaining adequate trajectory tracking effects for all axes. In 2010, Kato et al. [11] developed a novel, high-accuracy, fast-response pressure regulating valve for pneumatic vibration isolation tables by integrating the valve with active control. Chiang [12] developed X-Y servo pneumatic-piezoelectric hybrid actuators for position control with high response, large stroke and nanometer accuracy. In 2012, Lin incorporated a function approximation-based adaptive sliding mode with an H-inf tracking performance controller, applied this to three-axial pneumatic parallel manipulators, and achieved adequate trajectory tracking effects. In 2015, Antonio et al. [13] presented the sliding-mode control theory applied to analyze the dynamic behavior of the switching regulator and to establish the system stability conditions for a very high-voltage-gain single-stage boost converter operating at the boundary between continuous conduction mode (CCM) and discontinuous conduction mode (DCM). In 2016, Robinson et al. [14] applied adaptive neural network control to pneumatic artificial muscles and generated comprehensive and high-quality adaptive neural network control models that required little computational time when used under unknown pneumatic systems and joint dynamic model conditions. Shen and Sun proposed a nonlinear adaptive rotational speed control design and experiment of the propeller of an electric micro air vehicle. The hardware-in-the-loop of the experiments was set up for testing their validation [15]. Samet and Hasan [16] presented an optimized sliding mode control (SMC) strategy to maximize existence region for single-phase dynamic voltage restorers, and experimental results show the usefulness. Alessandro et al. [17] proposed that a sliding mode controller effectively combines a switched policy with a time-based adaptation of the control gain online adjusted. In 2017, Precup et al. [18] proposed two model-free sliding mode control system (MFSMCS) structures for the twin rotor aerodynamic system and the MFSMCS compared with a model-free intelligent proportional-integral (iPI) control system structure.

Compared with [19,20] which proposed mathematical modeling and control for pneumatic system simulations, the main aims of the present study is to implement and develop a hardware-in-the-loop system of a large-stroke asymmetric pneumatic servo system by incorporating sensing components for real-time positioning tracking servo control under variable loadings. A MATLAB Simulink real-time environment was successfully employed to set up the hardware-in-the-loop system for the closed-loop real-time path tracking servo control. In the experimental results, the maximum tracking errors for a large stroke is 0.222% better than those in references [21,22,23]. A dynamic analysis of the asymmetrical pneumatic system was performed, which included the derivation, analysis, and establishment of a mathematical model for the asymmetrical pneumatic system, as well as the design and analysis of an auxiliary controller. Sensors (i.e., position sensors) were then developed and incorporated into the prototype of this system, which comprised a large-stroke rod-less asymmetric pneumatic cylinder, pneumatic proportional servo valve, personal computer-based control system, controller algorithm, and data collection system. After the experimental system was designed and fully established, real-time control was performed by analyzing data collected from sensors to increase accurate positioning tracking performance for the large-stroke asymmetric pneumatic servo system.

## 2. The Sensor-Integrated Hardware-in-the-Loop of a Large-Stroke Asymmetric Pneumatic Servo System

**Figure 1:**
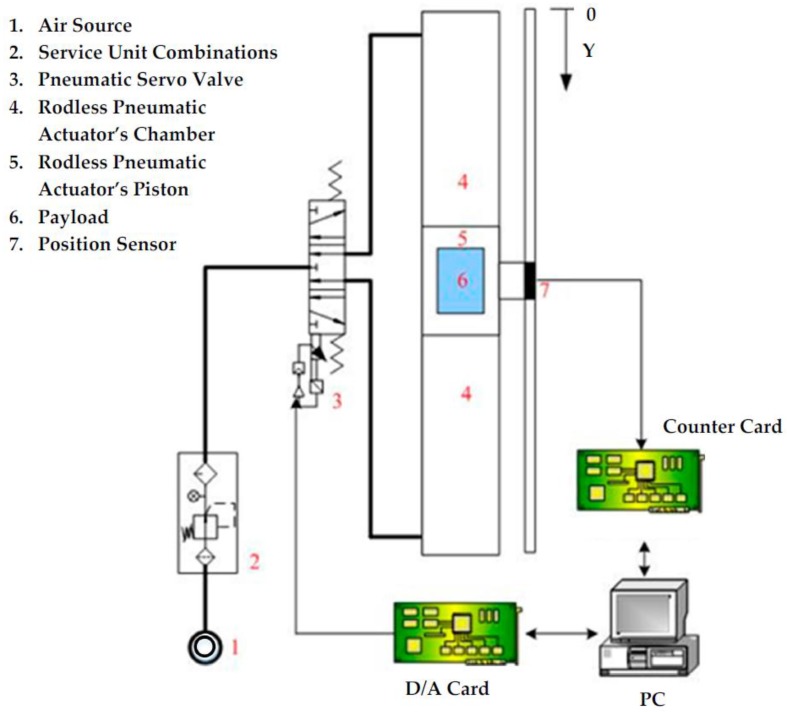
A sensor-integrated hardware-in-the-loop of a large-stroke asymmetric pneumatic servo system schema.

A sensor-integrated hardware-in-the-loop of a large-stroke asymmetric pneumatic servo system schema is shown in Figure 1, whose system is a sensor-integrated large-stroke asymmetric rod-less pneumatic servo system which has three main parts: a large-stroke asymmetric rod-less pneumatic system, a signal process system and a personal computer unit. For a large-stroke asymmetric rod-less pneumatic system, a rod-less pneumatic actuator with an asymmetrical load in the vertical y-axial direction is regulated by a pneumatic proportional directional control valve. Moreover, a signal process system consists of a linear optical encoder and data computing cards. Table 1 gives the specifications of the sensor-integrated large-stroke asymmetric pneumatic servo system. The rod-less pneumatic actuator, model DGC-40-1000 (FESTO AG, Esslingen, Germany), and the proportional directional control valve, model MPYE-5-1/4-010-B (FESTO AG, Esslingen, Germany), are considered in different tests. A linear optical encoder, with a resolution of 0.1 μm scale, is installed to measure the piston’s position. Also, the payload is 5 kg in the vertical y-axial direction.

**Figure 2:**
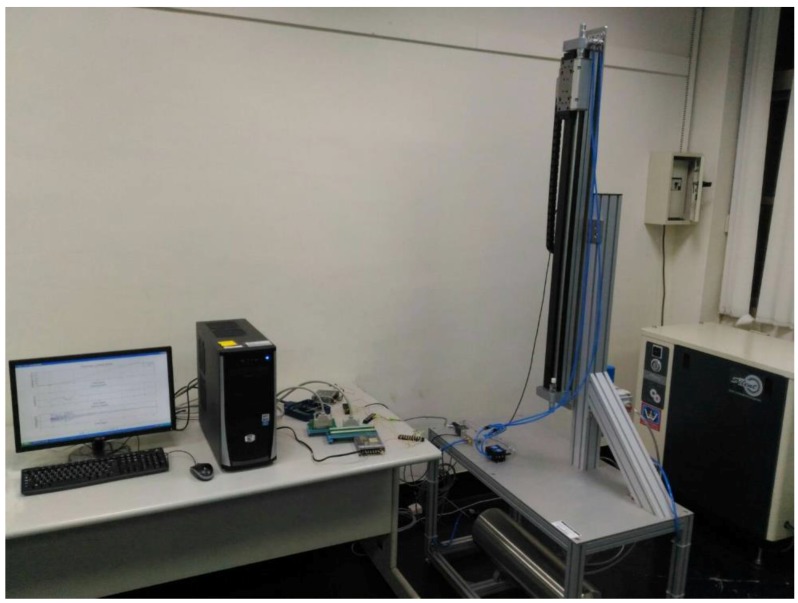
The system architecture of the proposed sensor-incorporated hardware-in-the-loop of a large-stroke asymmetric pneumatic servo system.

**Figure 3:**
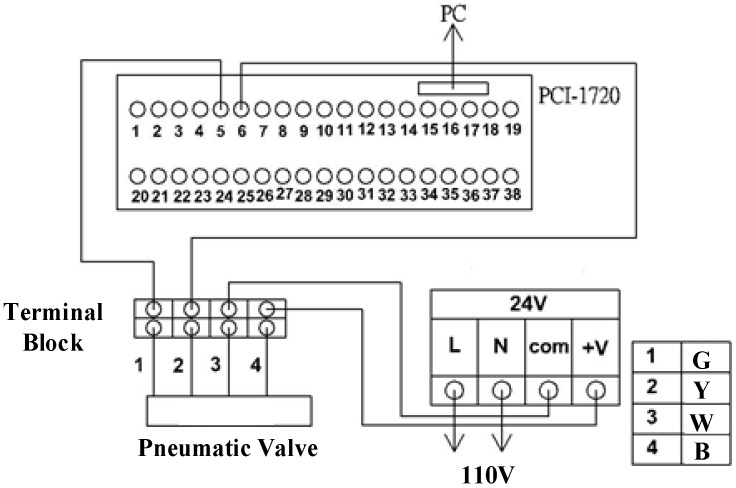
A wiring diagram of a PCI-1720 interface card.

Figure 2 presents the system architecture of the proposed sensor-integrated hardware-in-the-loop of a large-stroke asymmetric pneumatic servo system. In the PC-based control unit, a main computer unit (MCU) is responsible for processing the integrated digital/analogue processing system (DAPS) and a large-stroke asymmetric pneumatic servo system. The DAQ cards are installed and used to output the control signals and receive the input signal data from the optical encoder sensor. The control voltages of the proportional directional control valve are calculated by the real-time control algorithm via the MATLAB Simulink real-time environment in the computer and sent to the control valve via the analogue output channels on PCI-1720U DAQ (Advantech, Taiwan). Finally, the piston displacements of the rod-less cylinder measured by the linear encoder are counted and recorded by the counters on PCI-6601 DAQ card produced by National Instruments. Figure 3 illustrates a wiring diagram of PCI-1720 interface card. The present study established this system, designed a rod-less pneumatic cylinder, and observed its vertical movements, which result in asymmetric pressure on both sides of the pneumatic cylinder and asymmetric cylinder loads during axial motions. The function approximation technique was adopted for the design of an adaptive sliding mode control to perform a closed-loop system control. The MATLAB Simulink real-time environment is proposed to set up the overall hardware-in-the-loop system.

## 3. Establishment of Dynamic Models for the Pneumatic System

**Figure 4:**
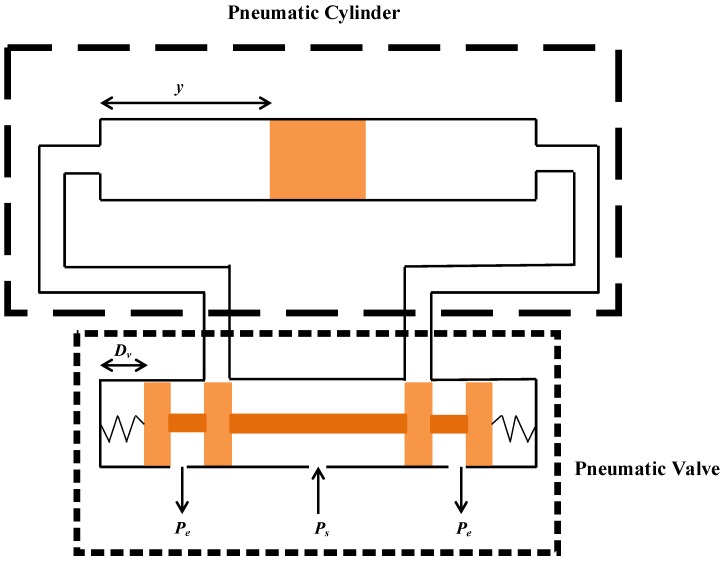
An architecture of a large stroke pneumatic system.

Figure 4 shows an architecture of a large stroke pneumatic system. In this study, the pneumatic system mainly consisted of a large rod-less pneumatic cylinder and a pneumatic proportional valve. The nonlinear dynamic models in the mathematic forms can be described in four parts: models of a pneumatic valve, mass flow rates of pneumatic cylinder, continuous equations and loading motion equations.

### 3.1. Models for the Pneumatic Valve

Models were developed for the pneumatic valve to describe the relationship between the control input voltage and displacements of the inner valve axis. The model can be expressed as a zero-, first-, or second-order model. Because the natural frequency of the servo valve was far higher than that of the pneumatic cylinder, the model of the pneumatic valve is expressed as a zero-order model using the following equation:

(1) $$D_{v}\left(t\right)=K_{v}u\left(t\right)$$

where $D_{v}\left(t\right)$ is the displacements of the inner valve axis, $K_{v}$ denotes the gain constant of valve axis displacement and control input voltage, and u(t) is a control input of a pneumatic valve.

If the gap between the valve axis and shift liner is not included, the relationship between valve axis displacement and the opening area of the servo valve can be expressed as:

(2) $$A_{o}\left(t\right)=K_{a}D_{v}\left(t\right)$$

where $A_{o}\left(t\right)$ denotes the opening area of the servo valve and $K_{a}$ denotes the relation coefficient between valve axis displacement and valve opening area.

### 3.2. Mass Flow Rates of a Pneumatic Cylinder

Mass flow rates of two chambers of a pneumatic cylinder (chambers A and B) is expressed:

(3) $$\dot{M_{a}}\left(t\right)=k_{1}A_{oa}\left(t\right)+k_{2}P_{a}\left(t\right)$$

(4) $$\dot{M_{b}}\left(t\right)=k_{1}A_{ob}\left(t\right)-k_{2}P_{b}\left(t\right)$$

where $\dot{M_{a}}\left(t\right)$ and $\dot{M_{b}}\left(t\right)$ are mass flow rates of chambers A and B respectively, $k_{1}=\frac{C_{d}C_{m}P_{i}}{\sqrt{T_{s}}}$ is gain for valve opening area and mass flow rate, $k_{2}=\frac{C_{d}C_{m}A_{oi}}{\sqrt{T_{s}}}$ is gain for pressure difference and mass flow rate, $C_{d}$ is displacement coefficient, $C_{m}$ is mass flow rate parameter, $P_{i}$ is work point pressure and $T_{s}$ is temperature of air supply.

In addition, the volume of Chambers A and B can be expressed as:

(5) $$V_{a}\left(t\right)=V_{i}+Ay\left(t\right)$$

(6) $$V_{b}\left(t\right)=V_{i}-Ay\left(t\right)$$

where $V_{i}$ is the initial volume including the volume of the pneumatic valve, the cylinder’s distribution pipes and the cylinder’s internal space. Also, y(t) is the piston displacement of the pneumatic cylinder.

### 3.3. Continuous Equations

Continuous equations consider the relationship between mass flow rate and pressure variation. The continuous equations for chambers A and B are shown:

(7) $$\dot{M_{a}}\left(t\right)=\frac{1}{RT_{1a}}\left(P_{a}\left(t\right)\frac{dV_{a}\left(t\right)}{dt}+\frac{V_{a}\left(t\right)}{r}\frac{dP_{a}\left(t\right)}{dt}\right)$$

(8) $$\dot{M_{b}}\left(t\right)=-\frac{1}{RT_{1b}}\left(P_{b}\left(t\right)\frac{dV_{b}\left(t\right)}{dt}+\frac{V_{b}\left(t\right)}{r}\frac{dP_{b}\left(t\right)}{dt}\right)$$

where R denotes the ideal gas constant, $T_{1a}$ and $T_{1b}$ denote temperatures of chambers A and B, $V_{a}\left(t\right)$ and $V_{b}\left(t\right)$ denote the volume of chambers A and B, and r denotes specific heat.

To simplify the analysis process, assume that the temperature of the proposed system always remains constant throughout the operation. Hence, $T_{1a}$ = $T_{1b}$ = $T_{s}$ leads to the following equation:

(9) $$\dot{M_{a}}\left(t\right)+\dot{M_{b}}\left(t\right)=\frac{1}{RT_{s}}\left(P_{a}\left(t\right)\frac{dV_{a}\left(t\right)}{dt}-P_{b}\left(t\right)\frac{dV_{b}\left(t\right)}{dt}+\frac{V_{a}\left(t\right)}{r}\dot{P_{a}}\left(t\right)-\frac{V_{b}\left(t\right)}{r}\dot{P_{b}}\left(t\right)\right)$$

### 3.4. The Load Motion Equation

By Newton’s second law of motion, the load motion equation is expressed as:

(10) $$A\left(P_{a}\left(t\right)-P_{b}\left(t\right)\right)sgn\left(\dot{y}\left(t\right)\right)=m\ddot{y}\left(t\right)+\mu_{u}\dot{y}\left(t\right)+ky\left(t\right)+\mu_{c}sgn\left(\dot{y}\left(t\right)\right)+f_{l}sgn\left(\dot{y}\left(t\right)\right)$$

where $\mu_{u}$ is a viscous friction coefficient, k is an elasticity coefficient, $\mu_{c}$ is a Coulomb friction force and $f_{l}$ is an external force.

Hence, the state equation for the pneumatic system can be derived by Equations (1)–(10) as follows:

(11) $$\{\begin{array}{c} \dot{x_{1}}\left(t\right)=x_{2}\left(t\right)\\ \dot{x_{2}}\left(t\right)=\frac{A\left(x_{3}\left(t\right)-x_{4}\left(t\right)\right)sgn\left(x_{2}\left(t\right)\right)-\mu_{u}x_{2}\left(t\right)-kx_{1}\left(t\right)-\mu_{c}sgn\left(x_{2}\left(t\right)\right)-f_{l}sgn\left(x_{2}\left(t\right)\right)}{m}\\ \dot{x_{3}}\left(t\right)=\frac{rRT_{1a}k_{1}k_{a}k_{v}u\left(t\right)+rRT_{1a}k_{2}x_{3}\left(t\right)-rx_{3}\left(t\right)Ax_{2}\left(t\right)}{Ax_{1}\left(t\right)+V_{i}}\\ \dot{x_{4}}\left(t\right)=\frac{-rRT_{1b}k_{1}k_{a}k_{v}u\left(t\right)+rRT_{1b}k_{2}x_{4}\left(t\right)+rx_{4}\left(t\right)Ax_{2}\left(t\right)}{V_{i}-Ax_{1}\left(t\right)} \end{array}$$

where $x_{1}\left(t\right)=y\left(t\right)$, $x_{2}\left(t\right)=\dot{y}\left(t\right)$, $x_{3}\left(t\right)=P_{a}\left(t\right)$, $x_{4}\left(t\right)=P_{b}\left(t\right)$.

## 4. Controller Design

**Figure 5:**
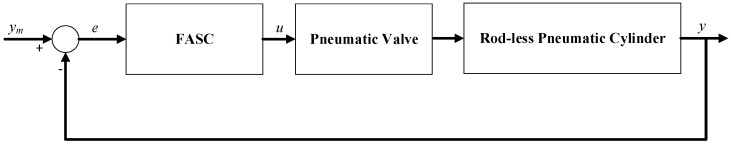
A system controller diagram.

As the proposed large-stroke asymmetric rod-less pneumatic system is a highly nonlinear system, an adaptive sliding mode controller was specially designed using a function approximation technique. The designed controller was used to solve the problems of system nonlinearity and time variation and thereby to control the large-stroke trajectory of the proposed system. The function approximation technique was the foundation of the mathematical models for the proposed system because such models are generally complex and accurate models can be difficult to obtain. Therefore, the functional approximation technique based on the sliding mode controller (FASC) is developed as a controller to solve the uncertain time-varying nonlinear system. The block diagram of a large stroke asymmetric rod-less pneumatic system is shown in Figure 5.

By Equation (11) discussed in Section 3, the pneumatic system can be expressed as

(12) $$\{\begin{array}{c} \dot{x}\left(t\right)=f\left(x,\;t\right)+g\left(x,\;t\right)u\left(t\right)\\ y\left(t\right)=h\left(x,\;t\right) \end{array}$$

where

$$x\left(t\right)=\lfloor \begin{array}{c} \begin{array}{c} x_{1}\left(t\right)\\ x_{2}\left(t\right)\\ x_{3}\left(t\right) \end{array}\\ x_{4}\left(t\right) \end{array}\rfloor$$

$$f\left(x,\;t\right)=\lfloor \begin{array}{c} x_{2}\left(t\right)\\ \frac{A\left(x_{3}\left(t\right)-x_{4}\left(t\right)\right)sgn\left(x_{2}\left(t\right)\right)-\mu_{u}x_{2}\left(t\right)-kx_{1}\left(t\right)-\mu_{c}sgn\left(x_{2}\left(t\right)\right)-f_{l}sgn\left(x_{2}\left(t\right)\right)}{m}\\ \frac{rRT_{1a}k_{2}x_{3}\left(t\right)-rx_{3}\left(t\right)Ax_{2}\left(t\right)}{Ax_{1}\left(t\right)+V_{i}}\\ \frac{rRT_{1b}k_{2}x_{4}\left(t\right)+rx_{4}\left(t\right)Ax_{2}\left(t\right)}{V_{i}-Ax_{1}\left(t\right)} \end{array}\rfloor$$

$$g\left(x,\;t\right)=\lfloor \begin{array}{c} \begin{array}{c} 0\\ 0\\ \frac{rRT_{1a}k_{1}k_{a}k_{v}}{Ax_{1}\left(t\right)+V_{i}} \end{array}\\ \frac{-rRT_{1b}k_{1}k_{a}k_{v}}{V_{i}-Ax_{1}\left(t\right)} \end{array}\rfloor$$

and $y\left(t\right)=x_{1}\left(t\right)$. f(x, t) and g(x, t) are unknown and smooth vector functions.

Thus, the dynamic models of the rod-less pneumatic system can be described as a nonlinear function shown as follows

(13) $$y^{\left(n\right)}\left(t\right)=F\left(x,\;t\right)+G\left(x,\;t\right)u\left(t\right)$$

where $x={\left[y\left(t\right)\;\dot{y}\left(t\right)\ldots \;y^{\left(n-1\right)}\left(t\right)\right]}^{T}\in R^{n}$ is the state vector, y(t)∈R is the output of the system. F(x, t) and G(x, t) are unknown time-varying function, and u(t)∈R is the control input of the system. The functional approximation technique is to approximate the functions F(x, t) and G(x, t).

An arbitrary function f(t) that falls within interval $\left[t_{1},\;t_{2}\right]$ can be expanded using orthogonal functions:

(14) $$f\left(t\right)=w_{1}q_{1}\left(t\right)+w_{2}q_{2}\left(t\right)+\ldots +w_{n}q_{n}\left(t\right)+\ldots$$

The present study employed Fourier series as the orthogonal function set. An arbitrary function f(t) that satisfies Dirichlet’s condition within interval $\left[t_{0},\;t_{0}+T\right]$ can be expanded as follows:

(15) $$f\left(t\right)=a_{0}+\overset{\infty }{\underset{n=1}{\sum }}[a_{n}cos\frac{2n\pi t}{T}+b_{n}sin\frac{2n\pi t}{T}]$$

where

$$\{\begin{array}{c} a_{0}=\frac{1}{T}\int_{t_{0}}^{t_{0}+T}f\left(t\right)dt\\ a_{n}=\frac{2}{T}\int_{t_{0}}^{t_{0}+T}f\left(t\right)cos\frac{2n\pi t}{T}dt\\ b_{n}=\frac{2}{T}\int_{t_{0}}^{t_{0}+T}f\left(t\right)sin\frac{2n\pi t}{T}dt \end{array}$$

Equation (15) denotes the Fourier series of f(t). $a_{0}$, $a_{n}$ and $b_{n}$ are coefficients of Fourier series. A function f(t) that satisfies Dirichlet’s condition can be approximated as follows:

(16) $$f\left(t\right)=\sum_{i=1}^{n}w_{i}z_{i}\left(t\right)+\epsilon \left(t\right)$$

where ϵ(t) denotes the truncation error.

If n is sufficiently large in Equation (16), then ϵ(t) can be ignored and f(t) can be approximated as the product of the coefficient vector and the orthogonal function vector:

(17) $$f\left(t\right)\approx W^{T}Z\left(t\right)$$

where $W={\left[w_{1}\;w_{2}\ldots \;w_{n}\right]}^{T}\;$ and $Z\left(t\right)={\left[z_{1}\left(t\right)\;z_{2}\left(t\right)\ldots \;z_{n}\left(t\right)\right]}^{T}$.

To ensure the system has an output of y(t), the control objective is adjusted with reference trajectory $y_{m}\left(t\right)$, with an output error that is expressed:

(18) $$e\left(t\right)=y\left(t\right)-y_{m}\left(t\right)$$

A sliding surface describes that as follows:

(19) $$s=a_{1}e\left(t\right)+a_{2}\dot{e}\left(t\right)+\ldots +e^{\left(n-1\right)}\left(t\right)$$

Therefore, the control output can be expressed as follows:

(20) $$u\left(t\right)=\frac{-{\hat{W_{F}}}^{T}Z_{F}\left(t\right)-\sum_{i=1}^{n-1}a_{i}e_{i+1}\left(t\right)-\sum_{i=1}^{n-1}p_{\left(n-1\right)i}e_{i}\left(t\right)+y_{m}^{\left(n\right)}\left(t\right)-\frac{s}{2\rho^{2}}}{{\hat{W_{g}}}^{T}Z_{g}\left(t\right)}$$

where ρ is a natural number.

In this study, the parameters of a controller obtained can be described as $s=a_{1}e\left(t\right)+a_{2}\dot{e}\left(t\right)+\ddot{e}\left(t\right)$ and $a_{1}=40$, $a_{2}=5$. Also, the initial values of Fourier coefficients $\hat{W_{F}}$ and $\hat{W_{g}}$ are ${\left[0,0,\ldots ,0\right]}_{1\times 11}$ and ${\left[20,000,0,\ldots ,0\right]}_{1\times 11}$. ρ is 0.2.

## 5. Experiments

### 5.1. The Hardware-in-the Loop of a Large-Stroke Asymmetric Pneumatic Servo System

**Figure 6:**
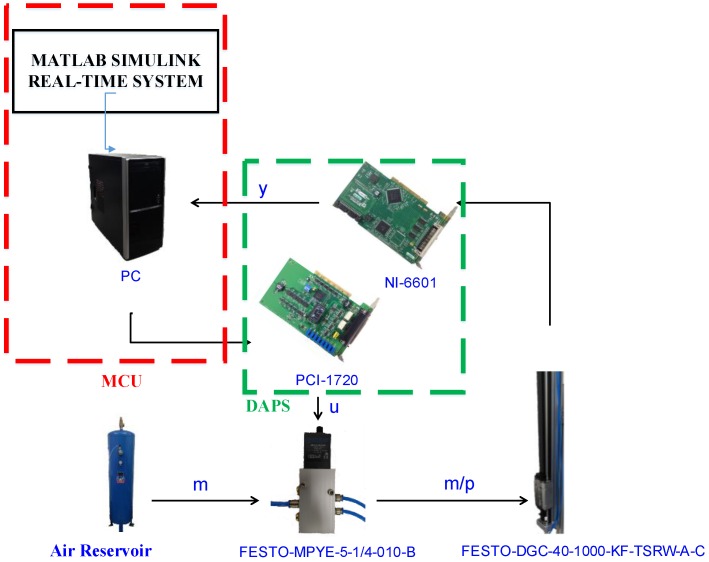
The hardware-in-the loop of a large-stroke asymmetric pneumatic servo system.

Figure 6 shows the hardware-in-the loop of a large-stroke asymmetric pneumatic servo system which consists of MCU, DAPS, a rod-less pneumatic cylinder, a proportional pneumatic valve and an air reservoir. The nonlinear controller, FASC, runs on PC via MATLAB Simulink real-time environment that can support reliable real-time control by digital I/O and analogy I/O for high precision and synchronization.

### 5.2. A Single Direction of 5th Path Real-Time Tracking Experiments

**Figure 7:**
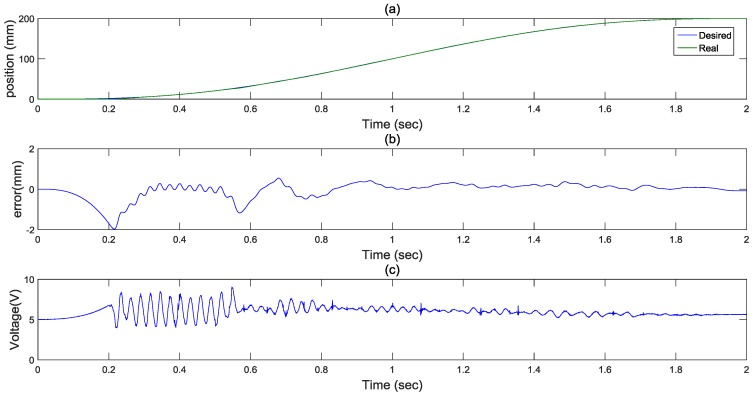
Experimental results for a 5th path with a stroke of 200 mm in 2 s: (**a**) position response (**b**) control error (**c**) control input.

The objective of this paper is to implement and realize a large-stroke asymmetric pneumatic servo system for real-time positioning tracking control. In the experiments, a 5th path is proposed for path tracking control in a small stroke and a large stroke. For a small stroke with a stroke of 200 mm in 2 s, the position responses, tracking errors and control inputs from FASC with a 5th path are shown in Figure 7. As can be seen in Figure 7b, tracking errors of the system can reach about 2 mm. The control inputs are shown in Figure 7c. Overall, the figure shows that a small path tracking control can be achieved.

**Figure 8:**
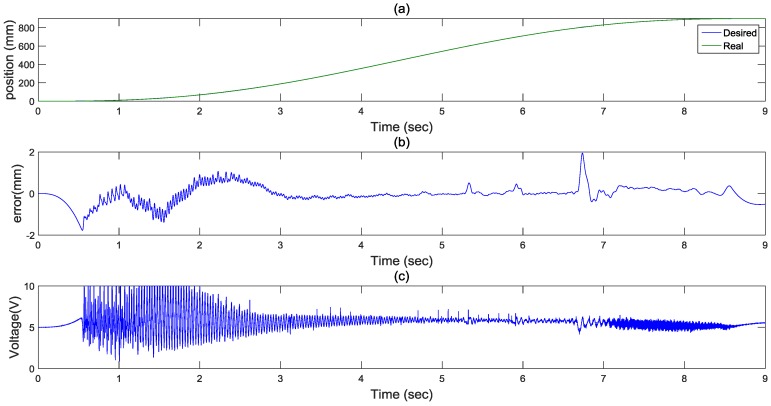
Experimental results for a 5th path with a stroke of 900 mm in 9 s: (**a**) position response (**b**) control error (**c**) control input.

For realization of path control in different strokes, a large stroke with a stroke of 900 mm in 9 s of the rod-less pneumatic system was implemented. Figure 8 shows the experimental results of path control by FASC for a 5th path tracking servo control. The biggest path control error is about 2 mm. Also, Figure 8c shows control signals of the pneumatic valve. Thus, the desired tracking performance of the FASC can be achieved for a large stroke.

### 5.3. A Bi-Direction of 5th Path Real-Time Tracking Experiments

**Figure 9:**
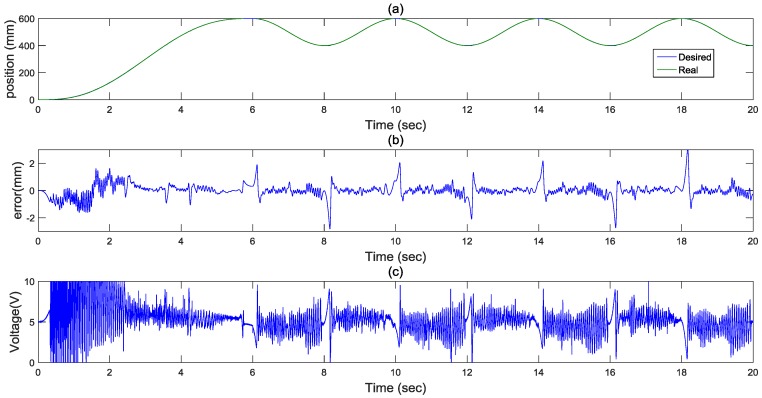
Experimental results for a sine wave path: (**a**) position response (**b**) control error (**c**) control input.

A sine wave function is proposed for bi-directional reciprocal motions. In order to confirm the bi-directional reciprocal motions, a sine wave with an amplitude of 100 mm and a period of $\frac{\pi }{2}\;$ rad is given and tested, as shown in Figure 9. First, the actuator moves along the 5th path for 600 mm for 6 s, and then moves along sine wave path for 14 s. As can be seen in Figure 9b, at the peak point of the sine wave where the motion direction changes, the path control error increases due to the nonlinearity of the friction force. Figure 9c show control inputs of the pneumatic valve. Therefore, the desired sine wave path can be achieved satisfactorily.

### 5.4. A Different Loading of 5th Path Real-Time Tracking Experiments

**Figure 10:**
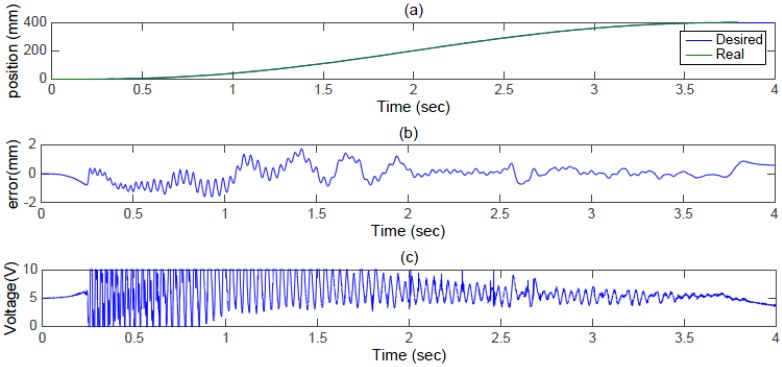
Experimental results for a 5th path with a stroke of 400 mm in 4 s under 45 degrees: (**a**) position response (**b**) control error (**c**) control input.

For realization of path tracking control in different payloads, a large stroke asymmetric pneumatic rod-less cylinder was set up in 45 degrees to consider different loading factors. Figure 10 shows the experimental results of a 5th path with a stroke of 400 mm in 4 s under 45 degrees. As can be seen in Figure 10b, the biggest path tracking control error is about 1.8 mm. The control inputs are shown in Figure 10c. For comparing the path tracking responses of different direction and loadings, Table 2 summarizes the tracking errors of the experiments.

Table 3 shows the comparison results of the FASC and relevant works in [21,22,23]. The maximum path tracking errors is defined as ${error}_{max}\left(\%\right)=\frac{Maximum}{Stroke}$. Compared with the relevant nonlinear controllers, the proposed FASC offers a better performance, with maximum errors of 0.222%, for the real-time path tracking servo system.

## 6. Conclusions

In this study, a hardware-in-the-loop of a large-stroke asymmetric pneumatic servo system by incorporating sensing components was developed and implemented for real-time path tracking control. A rod-less pneumatic actuator, a large stroke cylinder, was assigned in a vertical direction, where gravity effects render asymmetric motions. The mathematical models of a large-stroke asymmetric pneumatic system were analyzed in analytical forms. In order to implement the real-time control for a large-stroke asymmetric pneumatic servo system, the FASC was applied in the test rig based on MATLAB Simulink real-time environment. Afterwards, the experimental system was fully designed and established; the fifth path and the sine wave path are proposed to perform real-time control by analyzing data collected from sensor components to realize accurate positioning tracking performance for the large-stroke asymmetric pneumatic servo system. Also, different loading experiments under 45 degrees are verified experimentally. Compared with the relevant nonlinear controllers [21,22,23], the proposed FASC offers a better performance, with maximum errors of 0.222%, for the real-time path tracking servo system.

## Figures and Tables

**Table 1:** Specifications of the sensor-integrated hardware-in-the-loop of a large-stroke asymmetric pneumatic servo system.

Component	Specification
Pneumatic rod-less cylinder	Piston diameter: 40 mm
	Stroke: 1000 mm
Pneumatic proportional directional control valve	Valve function: 5/3 way
	Input voltage: 0–10 V
A/D D/A card	4-ch analog output with 12-bit D/A converter
Counter card	4-ch 32-bit counter with 20 MHz maximum source frequency
Optical encoder	Range: 1000 mm
	Resolution: 0.1 μm

**Table 2:** Comparison of the path tracking responses under different directions and loadings.

	Vertical Direction		45 Degrees Direction
	5th Path	Sine Wave	5th Path
Tracking Errors	2 mm	2 mm	1.8 mm

**Table 3:** Comparison of the functional approximation technique based on the sliding mode controller (FASC) and relevant works.

Controller	$Error_{max}$(%)
Ref. [19]	0.599
Ref. [20]	1.6
Ref. [21]	1.25
**FASC**	0.222
